# Crystal structure of (4*Z*)-4-[(2*E*)-3-(2-chloro­phen­yl)-1-hy­droxy­prop-2-en-1-yl­idene]-3-methyl-1-phenyl-1*H*-pyrazol-5(4*H*)-one

**DOI:** 10.1107/S2056989015009020

**Published:** 2015-05-20

**Authors:** Muhammad Shahid, Munawar Ali Munawar, Muhammad Nawaz Tahir, Muhammad Salim, Khizar Iqbal Malik

**Affiliations:** a Department of Chemistry, University of the Punjab, Lahore, Punjab, Pakistan; b Department of Physics, University of Sargodha, Sargodha, Punjab, Pakistan

**Keywords:** crystal structure, pyrazole, hydrogen bonding, π–π inter­actions

## Abstract

In the title compound, C_19_H_15_ClN_2_O_2_, the pyrazole ring is almost planar (r.m.s. deviation = 0.002 Å) and subtends dihedral angles of 5.31 (16) and 1.86 (16)° with the phenyl and chloro­benzene rings, respectively. An intra­molecular O—H⋯O hydrogen bond closes an *S*(6) ring and a short C—H⋯O contact is also observed. In the crystal, mol­ecules are linked by weak C—H⋯O inter­actions to generate (001) sheets. Weak aromatic π–π inter­actions between the chloro­benzene and pyrazole rings, with a centroid–centroid distance of 3.7956 (17) Å are also observed.

## Related literature   

For related structures, see: Chaudhry *et al.* (2012 ▸); Holzer *et al.* (1999 ▸); Malik *et al.* (2009 ▸).
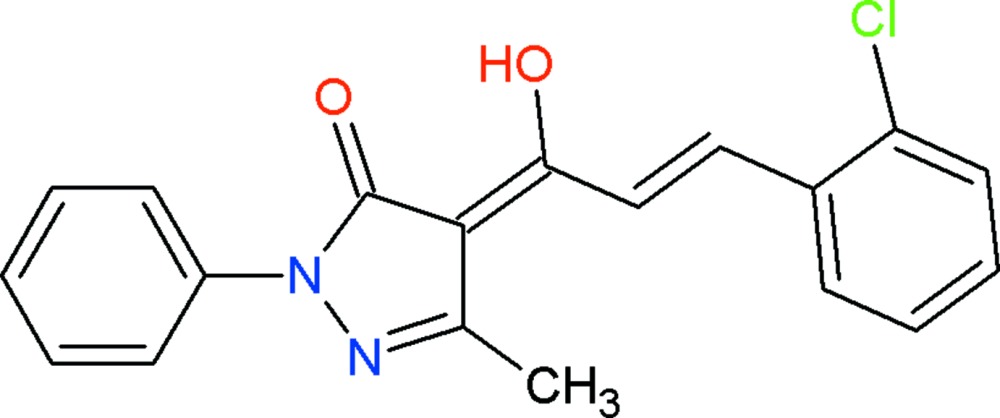


## Experimental   

### Crystal data  

C_19_H_15_ClN_2_O_2_
*M* *_r_* = 338.78
Orthorhombic, 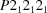

*a* = 7.2348 (3) Å
*b* = 12.8737 (6) Å
*c* = 17.7843 (7) Å
*V* = 1656.41 (12) Å^3^
*Z* = 4
Mo *K*α radiation
μ = 0.24 mm^−1^
*T* = 296 K
0.34 × 0.28 × 0.16 mm

### Data collection  

Bruker Kappa APEXII CCD diffractometer
Absorption correction: multi-scan (*SADABS*; Bruker, 2005 ▸) *T*_min_ = 0.923, *T*_max_ = 0.960
8199 measured reflections
3593 independent reflections
2455 reflections with *I* > 2σ(*I*)
*R*_int_ = 0.034

### Refinement  

*R*[*F* ^2^ > 2σ(*F* ^2^)] = 0.043
*wR*(*F* ^2^) = 0.091
*S* = 1.00
3593 reflections
219 parameters
H-atom parameters constrained
Δρ_max_ = 0.13 e Å^−3^
Δρ_min_ = −0.17 e Å^−3^
Absolute structure: Flack *x* determined using 771 quotients [(*I* ^+^)−(*I* ^−^)]/[(*I* ^+^)+(*I* ^−^)] (Parsons *et al.*, 2013 ▸)
Absolute structure parameter: −0.06 (4)

### 

Data collection: *APEX2* (Bruker, 2007 ▸); cell refinement: *SAINT* (Bruker, 2007 ▸); data reduction: *SAINT*; program(s) used to solve structure: *SHELXS97* (Sheldrick, 2008 ▸); program(s) used to refine structure: *SHELXL2014* (Sheldrick, 2015 ▸); molecular graphics: *ORTEP-3 for Windows* (Farrugia, 2012 ▸) and *PLATON* (Spek, 2009 ▸); software used to prepare material for publication: *WinGX* (Farrugia, 2012 ▸) and *PLATON*.

## Supplementary Material

Crystal structure: contains datablock(s) global, I. DOI: 10.1107/S2056989015009020/hb7419sup1.cif

Structure factors: contains datablock(s) I. DOI: 10.1107/S2056989015009020/hb7419Isup2.hkl

Supporting information file. DOI: 10.1107/S2056989015009020/hb7419Isup3.cml

**Fig. 1.**
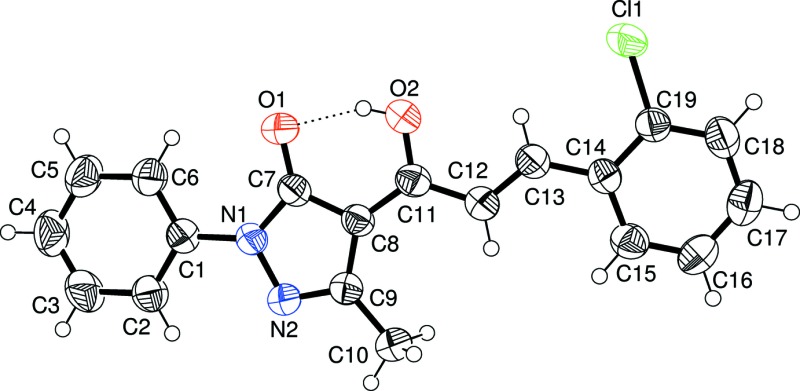
View of the title compound with displacement ellipsoids drawn at the 50% probability level.

. DOI: 10.1107/S2056989015009020/hb7419fig1.tif
View of the title compound with displacement ellipsoids drawn at the 50% probability level.

**Fig. 2.**
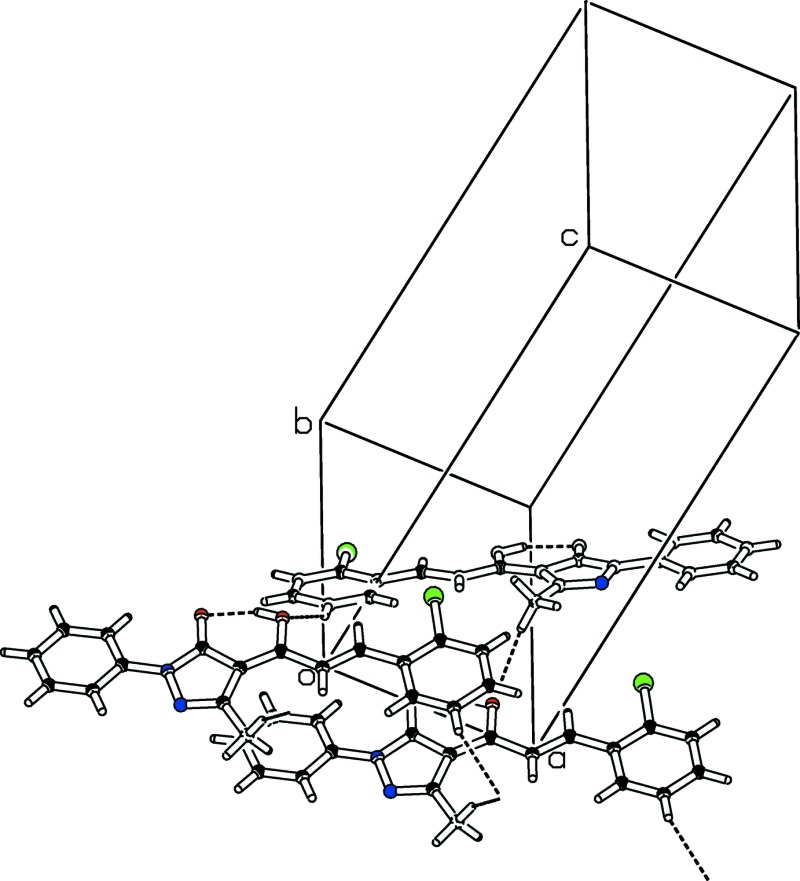
The partial packing, which shows that molecules are interlinked due to O—H···O bondings.

. DOI: 10.1107/S2056989015009020/hb7419fig2.tif
The partial packing, which shows that mol­ecules are inter­linked due to O—H⋯O bondings.

CCDC reference: 1400008

Additional supporting information:  crystallographic information; 3D view; checkCIF report

## Figures and Tables

**Table 1:** Hydrogen-bond geometry (, )

*D*H*A*	*D*H	H*A*	*D* *A*	*D*H*A*
O2H2*A*O1	0.82	1.74	2.501(3)	154
C6H6O1	0.93	2.29	2.933(4)	126
C10H10*B*O2^i^	0.96	2.55	3.444(4)	155
C16H16O2^ii^	0.93	2.56	3.405(4)	151

Symmetry codes: (i) 
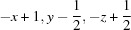
; (ii) 
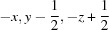
.

## supplementary crystallographic information

### S1. Comment

The crystal structures of 5-methyl-2-phenyl-4-((*E*)-3-phenyl-2-hydroxy- prop-2-enylidene)-1,2-dihydro-3*H*-pyrazol-3-one (Holzer *et al.*, 1999), (4*Z*)-4-((2*E*)-1-hydroxy-3-(4-methoxyphenyl)prop-2-en-1- ylidene)-3-methyl-1-phenyl-1*H*-pyrazol-5(4*H*)-one (Malik *et al.*, 2009) and (4*Z*)-4-((2*E*)-1-hydroxy-3-(3-nitrophenyl)prop- 2-en-1-ylidene)-3-methyl-1-(4-methylphenyl)-1*H*-pyrazol-5(4*H*)-one (Chaudhry, *et al.*, 2012) have been published which are related to the title compound (I, Fig. 1). (I) is synthesized for the biological studies as well as for the preparation of different metal complexes.

In (I), the benzene ring A (C1–C6) and the 4-[(2*E*)-3-(2-chlorophenyl)-1- hydroxyprop-2-en-1-ylidene]-5-methyl-2,4-dihydro-3*H*-pyrazol-3-one moiety (C7 –C19/N1/N2/O1/O2/CL1) are planar with r. m. s. deviation of 0.0016 and 0.0158 Å, respectively. The dihedral angle between A/B is 4.87 (14)°. There exist intramolecular H-bonding of O—H···O type completing *S* (6) loop (Bernstein *et al.*, 1995). The molecules are interlimked due to C—H···O interactions (Table 1, Fig. 2). There exist π–π interactions at a distance of 3.7956 (17) Å between the centeroids of *Cg*1—*Cg*2^i^ and *Cg*2— *Cg*1^ii^ [i = 1 + *x*, *y*, *z* and ii = -1 + *x*, *y*, *z*], where *Cg*1 and *Cg*2 are the centroids of heterocyclic ring *C* (N1/N2/C7/C8/C9) and chloro containing benzene ring *D* (C14–C19), respectively.

### S2. Experimental

4-Acetyl-3-methyl-1-phenyl-5-hydroxypyrazole (0.218 g, 1 mmol), 2-chlorobenzaldehyde (0.211 g, 1.5 mmol) in glacial acetic acid (10 ml) and concentrated sulfuric acid (0.2 ml) was stirred at 353–360 K for 6 h. The reaction mixture was diluted with distilled water (50 ml). The precipitate was filtered, washed with methanol and dried. The crude product was purified by column chromatography using n-hexane and ethyl acetate mixtures as eluents. The product was recrystallized from n-hexane solution to afford yellow plates. Yield = 60%; m.p. 453 K

### S3. Refinement

The H atoms were positioned geometrically (C–H = 0.93–0.96 Å, O—H= 0.82 Å) and refined as riding with *U*_iso_(H) = *xU*_eq_(C, O), where *x* = 1.5 for methyl and hydroxy and *x* =1.2 for other H-atoms.

### Crystal data

**Table:**

C_19_H_15_ClN_2_O_2_	*D*_x_ = 1.358 Mg m^−^^3^
*M**_r_* = 338.78	Mo *K*α radiation, λ = 0.71073 Å
Orthorhombic, *P*2_1_2_1_2_1_	Cell parameters from 2455 reflections
*a* = 7.2348 (3) Å	θ = 2.3–27.0°
*b* = 12.8737 (6) Å	µ = 0.24 mm^−^^1^
*c* = 17.7843 (7) Å	*T* = 296 K
*V* = 1656.41 (12) Å^3^	Plate, yellow
*Z* = 4	0.34 × 0.28 × 0.16 mm
*F*(000) = 704	

### Data collection

**Table:**

Bruker Kappa APEXII CCD diffractometer	3593 independent reflections
Radiation source: fine-focus sealed tube	2455 reflections with *I* > 2σ(*I*)
Graphite monochromator	*R*_int_ = 0.034
Detector resolution: 7.70 pixels mm^-1^	θ_max_ = 27.0°, θ_min_ = 2.3°
ω scans	*h* = −8→9
Absorption correction: multi-scan (*SADABS*; Bruker, 2005)	*k* = −16→13
*T*_min_ = 0.923, *T*_max_ = 0.960	*l* = −22→20
8199 measured reflections	

### Refinement

**Table:**

Refinement on *F*^2^	Secondary atom site location: difference Fourier map
Least-squares matrix: full	Hydrogen site location: inferred from neighbouring sites
*R*[*F*^2^ > 2σ(*F*^2^)] = 0.043	H-atom parameters constrained
*wR*(*F*^2^) = 0.091	*w* = 1/[σ^2^(*F*_o_^2^) + (0.0369*P*)^2^] where *P* = (*F*_o_^2^ + 2*F*_c_^2^)/3
*S* = 1.00	(Δ/σ)_max_ < 0.001
3593 reflections	Δρ_max_ = 0.13 e Å^−^^3^
219 parameters	Δρ_min_ = −0.17 e Å^−^^3^
0 restraints	Absolute structure: Flack *x* determined using 771 quotients [(*I*^+^)-(*I*^-^)]/[(*I*^+^)+(*I*^-^)] (Parsons *et al.*, 2013)
Primary atom site location: structure-invariant direct methods	Absolute structure parameter: −0.06 (4)

### Special details

**Table:**

Geometry. All e.s.d.'s (except the e.s.d. in the dihedral angle between two l.s. planes) are estimated using the full covariance matrix. The cell e.s.d.'s are taken into account individually in the estimation of e.s.d.'s in distances, angles and torsion angles; correlations between e.s.d.'s in cell parameters are only used when they are defined by crystal symmetry. An approximate (isotropic) treatment of cell e.s.d.'s is used for estimating e.s.d.'s involving l.s. planes.
Refinement. Refinement of *F*^2^ against ALL reflections. The weighted *R*-factor *wR* and goodness of fit *S* are based on *F*^2^, conventional *R*-factors *R* are based on *F*, with *F* set to zero for negative *F*^2^. The threshold expression of *F*^2^ > σ(*F*^2^) is used only for calculating *R*-factors(gt) *etc*. and is not relevant to the choice of reflections for refinement. *R*-factors based on *F*^2^ are statistically about twice as large as those based on *F*, and *R*-factors based on ALL data will be even larger.

### Fractional atomic coordinates and isotropic or equivalent isotropic displacement parameters (Å^2^)

**Table:**

	*x*	*y*	*z*	*U*_iso_*/*U*_eq_	
C1	1.0397 (4)	0.0392 (2)	0.44274 (15)	0.0467 (7)	
C2	1.1377 (5)	−0.0394 (3)	0.47830 (17)	0.0585 (9)	
H2	1.0927	−0.1070	0.4786	0.070*	
C3	1.3019 (5)	−0.0158 (3)	0.5130 (2)	0.0757 (11)	
H3	1.3684	−0.0684	0.5366	0.091*	
C4	1.3695 (5)	0.0835 (4)	0.51358 (19)	0.0788 (11)	
H4	1.4806	0.0983	0.5376	0.095*	
C5	1.2721 (5)	0.1611 (3)	0.47842 (19)	0.0754 (11)	
H5	1.3174	0.2287	0.4786	0.091*	
C6	1.1075 (5)	0.1391 (3)	0.44288 (18)	0.0612 (9)	
H6	1.0421	0.1918	0.4190	0.073*	
C7	0.7566 (4)	0.0745 (2)	0.36469 (16)	0.0472 (7)	
C8	0.6056 (4)	0.0103 (2)	0.34325 (16)	0.0454 (7)	
C9	0.6447 (4)	−0.0886 (2)	0.37664 (16)	0.0495 (7)	
C10	0.5360 (5)	−0.1872 (2)	0.37362 (19)	0.0705 (10)	
H10A	0.5947	−0.2387	0.4045	0.106*	
H10B	0.5305	−0.2115	0.3226	0.106*	
H10C	0.4130	−0.1747	0.3918	0.106*	
C11	0.4645 (4)	0.0533 (2)	0.29952 (16)	0.0492 (8)	
C12	0.3000 (4)	−0.0007 (3)	0.27371 (16)	0.0512 (8)	
H12	0.2835	−0.0702	0.2864	0.061*	
C13	0.1726 (4)	0.0468 (3)	0.23235 (17)	0.0507 (8)	
H13	0.1955	0.1162	0.2212	0.061*	
C14	0.0018 (4)	0.0040 (2)	0.20232 (15)	0.0455 (8)	
C15	−0.0492 (5)	−0.0993 (3)	0.21263 (16)	0.0572 (8)	
H15	0.0281	−0.1428	0.2400	0.069*	
C16	−0.2102 (5)	−0.1386 (3)	0.18351 (18)	0.0649 (10)	
H16	−0.2405	−0.2080	0.1912	0.078*	
C17	−0.3274 (5)	−0.0754 (3)	0.1427 (2)	0.0672 (10)	
H17	−0.4368	−0.1020	0.1232	0.081*	
C18	−0.2818 (5)	0.0269 (3)	0.13120 (18)	0.0625 (9)	
H18	−0.3598	0.0699	0.1036	0.075*	
C19	−0.1203 (4)	0.0655 (2)	0.16072 (15)	0.0481 (8)	
Cl1	−0.06694 (12)	0.19522 (6)	0.14234 (5)	0.0687 (3)	
N1	0.8699 (3)	0.01404 (19)	0.40719 (13)	0.0472 (6)	
N2	0.7992 (4)	−0.0870 (2)	0.41388 (14)	0.0539 (7)	
O1	0.7814 (3)	0.16964 (16)	0.34743 (13)	0.0638 (6)	
O2	0.4797 (3)	0.15046 (17)	0.28011 (14)	0.0659 (7)	
H2A	0.5725	0.1754	0.2995	0.099*	

### Atomic displacement parameters (Å^2^)

**Table:**

	*U*^11^	*U*^22^	*U*^33^	*U*^12^	*U*^13^	*U*^23^
C1	0.0501 (18)	0.0511 (19)	0.0389 (15)	0.0025 (16)	−0.0027 (14)	−0.0056 (14)
C2	0.065 (2)	0.060 (2)	0.0516 (18)	0.0003 (18)	−0.0134 (17)	0.0015 (16)
C3	0.078 (3)	0.083 (3)	0.065 (2)	0.009 (2)	−0.030 (2)	0.005 (2)
C4	0.067 (2)	0.097 (3)	0.072 (2)	−0.009 (2)	−0.028 (2)	0.000 (2)
C5	0.076 (3)	0.075 (3)	0.076 (2)	−0.018 (2)	−0.020 (2)	−0.003 (2)
C6	0.063 (2)	0.059 (2)	0.062 (2)	−0.0041 (17)	−0.0131 (18)	−0.0029 (17)
C7	0.0489 (18)	0.0464 (19)	0.0461 (16)	0.0063 (15)	−0.0043 (15)	−0.0001 (15)
C8	0.0464 (18)	0.0436 (18)	0.0463 (16)	0.0051 (14)	−0.0026 (14)	−0.0017 (14)
C9	0.0484 (18)	0.0452 (18)	0.0549 (18)	0.0022 (15)	−0.0027 (15)	−0.0021 (15)
C10	0.071 (2)	0.048 (2)	0.093 (3)	−0.0056 (19)	−0.021 (2)	0.0053 (19)
C11	0.0522 (19)	0.0455 (19)	0.0500 (17)	0.0059 (16)	0.0014 (15)	−0.0019 (14)
C12	0.0507 (19)	0.049 (2)	0.0538 (17)	0.0027 (16)	−0.0040 (16)	0.0020 (16)
C13	0.049 (2)	0.051 (2)	0.0522 (17)	0.0037 (16)	−0.0020 (15)	0.0016 (15)
C14	0.0431 (18)	0.051 (2)	0.0422 (15)	0.0051 (15)	0.0016 (13)	−0.0021 (14)
C15	0.060 (2)	0.056 (2)	0.0559 (19)	0.0020 (19)	0.0010 (17)	0.0079 (16)
C16	0.067 (2)	0.060 (2)	0.068 (2)	−0.015 (2)	0.007 (2)	−0.0019 (18)
C17	0.051 (2)	0.079 (3)	0.072 (2)	−0.009 (2)	0.0011 (18)	−0.016 (2)
C18	0.053 (2)	0.070 (2)	0.064 (2)	0.0112 (18)	−0.0118 (17)	−0.0112 (19)
C19	0.0488 (18)	0.045 (2)	0.0500 (17)	0.0073 (15)	0.0004 (15)	−0.0081 (15)
Cl1	0.0766 (6)	0.0463 (5)	0.0832 (6)	0.0147 (5)	−0.0178 (5)	−0.0019 (5)
N1	0.0470 (15)	0.0445 (16)	0.0502 (14)	0.0025 (12)	−0.0078 (12)	−0.0009 (12)
N2	0.0547 (16)	0.0438 (16)	0.0631 (17)	−0.0022 (14)	−0.0102 (13)	0.0033 (13)
O1	0.0687 (14)	0.0432 (13)	0.0794 (15)	−0.0022 (11)	−0.0151 (13)	0.0083 (12)
O2	0.0594 (15)	0.0523 (15)	0.0860 (17)	0.0026 (11)	−0.0196 (13)	0.0104 (13)

### Geometric parameters (Å, º)

**Table:**

C1—C6	1.377 (4)	C10—H10C	0.9600
C1—C2	1.387 (4)	C11—O2	1.302 (3)
C1—N1	1.419 (4)	C11—C12	1.453 (4)
C2—C3	1.372 (4)	C12—C13	1.328 (4)
C2—H2	0.9300	C12—H12	0.9300
C3—C4	1.370 (5)	C13—C14	1.454 (4)
C3—H3	0.9300	C13—H13	0.9300
C4—C5	1.373 (5)	C14—C15	1.392 (4)
C4—H4	0.9300	C14—C19	1.398 (4)
C5—C6	1.377 (4)	C15—C16	1.372 (5)
C5—H5	0.9300	C15—H15	0.9300
C6—H6	0.9300	C16—C17	1.381 (5)
C7—O1	1.276 (3)	C16—H16	0.9300
C7—N1	1.360 (3)	C17—C18	1.373 (5)
C7—C8	1.421 (4)	C17—H17	0.9300
C8—C11	1.397 (4)	C18—C19	1.374 (4)
C8—C9	1.433 (4)	C18—H18	0.9300
C9—N2	1.299 (4)	C19—Cl1	1.745 (3)
C9—C10	1.494 (4)	N1—N2	1.403 (3)
C10—H10A	0.9600	O2—H2A	0.8200
C10—H10B	0.9600		
			
C6—C1—C2	119.9 (3)	O2—C11—C8	117.8 (3)
C6—C1—N1	121.5 (3)	O2—C11—C12	116.4 (3)
C2—C1—N1	118.6 (3)	C8—C11—C12	125.8 (3)
C3—C2—C1	119.1 (3)	C13—C12—C11	121.6 (3)
C3—C2—H2	120.4	C13—C12—H12	119.2
C1—C2—H2	120.4	C11—C12—H12	119.2
C4—C3—C2	121.2 (4)	C12—C13—C14	128.2 (3)
C4—C3—H3	119.4	C12—C13—H13	115.9
C2—C3—H3	119.4	C14—C13—H13	115.9
C3—C4—C5	119.5 (4)	C15—C14—C19	116.3 (3)
C3—C4—H4	120.2	C15—C14—C13	122.6 (3)
C5—C4—H4	120.2	C19—C14—C13	121.1 (3)
C4—C5—C6	120.2 (4)	C16—C15—C14	121.9 (3)
C4—C5—H5	119.9	C16—C15—H15	119.1
C6—C5—H5	119.9	C14—C15—H15	119.1
C1—C6—C5	120.0 (3)	C15—C16—C17	120.1 (3)
C1—C6—H6	120.0	C15—C16—H16	119.9
C5—C6—H6	120.0	C17—C16—H16	119.9
O1—C7—N1	126.8 (3)	C18—C17—C16	119.8 (3)
O1—C7—C8	127.0 (3)	C18—C17—H17	120.1
N1—C7—C8	106.3 (3)	C16—C17—H17	120.1
C11—C8—C7	118.8 (3)	C17—C18—C19	119.6 (3)
C11—C8—C9	136.6 (3)	C17—C18—H18	120.2
C7—C8—C9	104.7 (3)	C19—C18—H18	120.2
N2—C9—C8	111.5 (3)	C18—C19—C14	122.4 (3)
N2—C9—C10	118.9 (3)	C18—C19—Cl1	117.5 (2)
C8—C9—C10	129.5 (3)	C14—C19—Cl1	120.1 (2)
C9—C10—H10A	109.5	C7—N1—N2	111.0 (2)
C9—C10—H10B	109.5	C7—N1—C1	129.7 (3)
H10A—C10—H10B	109.5	N2—N1—C1	119.3 (2)
C9—C10—H10C	109.5	C9—N2—N1	106.5 (2)
H10A—C10—H10C	109.5	C11—O2—H2A	109.5
H10B—C10—H10C	109.5		
			
C6—C1—C2—C3	−0.2 (5)	C12—C13—C14—C19	178.7 (3)
N1—C1—C2—C3	−179.9 (3)	C19—C14—C15—C16	0.1 (4)
C1—C2—C3—C4	0.5 (5)	C13—C14—C15—C16	−179.6 (3)
C2—C3—C4—C5	−0.4 (6)	C14—C15—C16—C17	−0.1 (5)
C3—C4—C5—C6	0.0 (6)	C15—C16—C17—C18	0.3 (5)
C2—C1—C6—C5	−0.1 (5)	C16—C17—C18—C19	−0.3 (5)
N1—C1—C6—C5	179.5 (3)	C17—C18—C19—C14	0.3 (5)
C4—C5—C6—C1	0.2 (5)	C17—C18—C19—Cl1	178.5 (2)
O1—C7—C8—C11	−1.1 (5)	C15—C14—C19—C18	−0.2 (4)
N1—C7—C8—C11	179.1 (2)	C13—C14—C19—C18	179.5 (3)
O1—C7—C8—C9	179.7 (3)	C15—C14—C19—Cl1	−178.3 (2)
N1—C7—C8—C9	−0.1 (3)	C13—C14—C19—Cl1	1.3 (4)
C11—C8—C9—N2	−179.1 (3)	O1—C7—N1—N2	−179.5 (3)
C7—C8—C9—N2	−0.1 (3)	C8—C7—N1—N2	0.3 (3)
C11—C8—C9—C10	1.1 (6)	O1—C7—N1—C1	0.2 (5)
C7—C8—C9—C10	−179.8 (3)	C8—C7—N1—C1	−180.0 (2)
C7—C8—C11—O2	0.5 (4)	C6—C1—N1—C7	5.6 (5)
C9—C8—C11—O2	179.4 (3)	C2—C1—N1—C7	−174.8 (3)
C7—C8—C11—C12	−179.1 (3)	C6—C1—N1—N2	−174.8 (3)
C9—C8—C11—C12	−0.2 (5)	C2—C1—N1—N2	4.9 (4)
O2—C11—C12—C13	0.2 (5)	C8—C9—N2—N1	0.3 (3)
C8—C11—C12—C13	179.8 (3)	C10—C9—N2—N1	−179.9 (2)
C11—C12—C13—C14	180.0 (3)	C7—N1—N2—C9	−0.4 (3)
C12—C13—C14—C15	−1.7 (5)	C1—N1—N2—C9	179.9 (2)

### Hydrogen-bond geometry (Å, º)

**Table:**

*D*—H···*A*	*D*—H	H···*A*	*D*···*A*	*D*—H···*A*
O2—H2*A*···O1	0.82	1.74	2.501 (3)	154
C6—H6···O1	0.93	2.29	2.933 (4)	126
C10—H10*B*···O2^i^	0.96	2.55	3.444 (4)	155
C16—H16···O2^ii^	0.93	2.56	3.405 (4)	151

Symmetry codes: (i) −*x*+1, *y*−1/2, −*z*+1/2; (ii) −*x*, *y*−1/2, −*z*+1/2.
