# Supplementary figures and images for: Crystal structure of (4Z)-4-[(2E)-3-(2-chloro­phen­yl)-1-hy­droxy­prop-2-en-1-yl­idene]-3-methyl-1-phenyl-1H-pyrazol-5(4H)-one
Source: Acta Crystallogr E Crystallogr Commun. 2015 May 20;71(Pt 6):o407–8. doi: 10.1107/S2056989015009020 (PMC4459312; doi:10.1107/S2056989015009020)

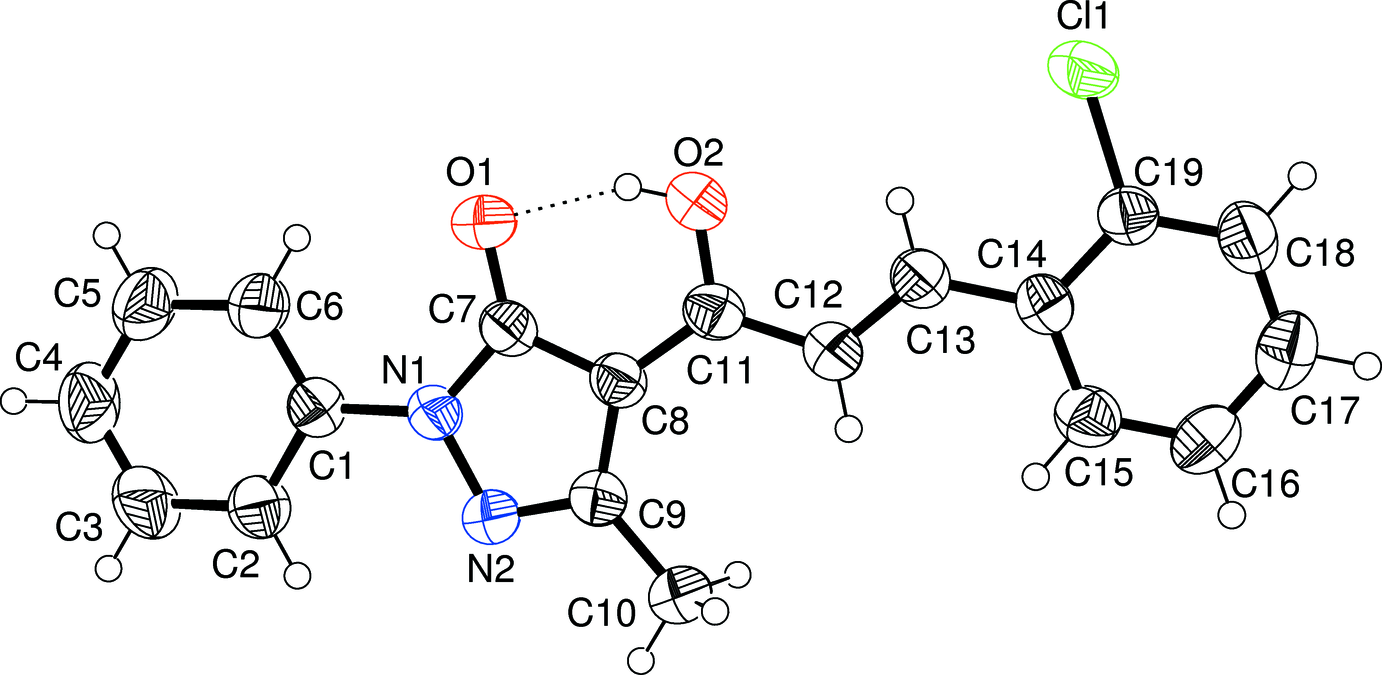

Supplement: Supplementary file 4 [file e-71-0o407-fig1.tif]

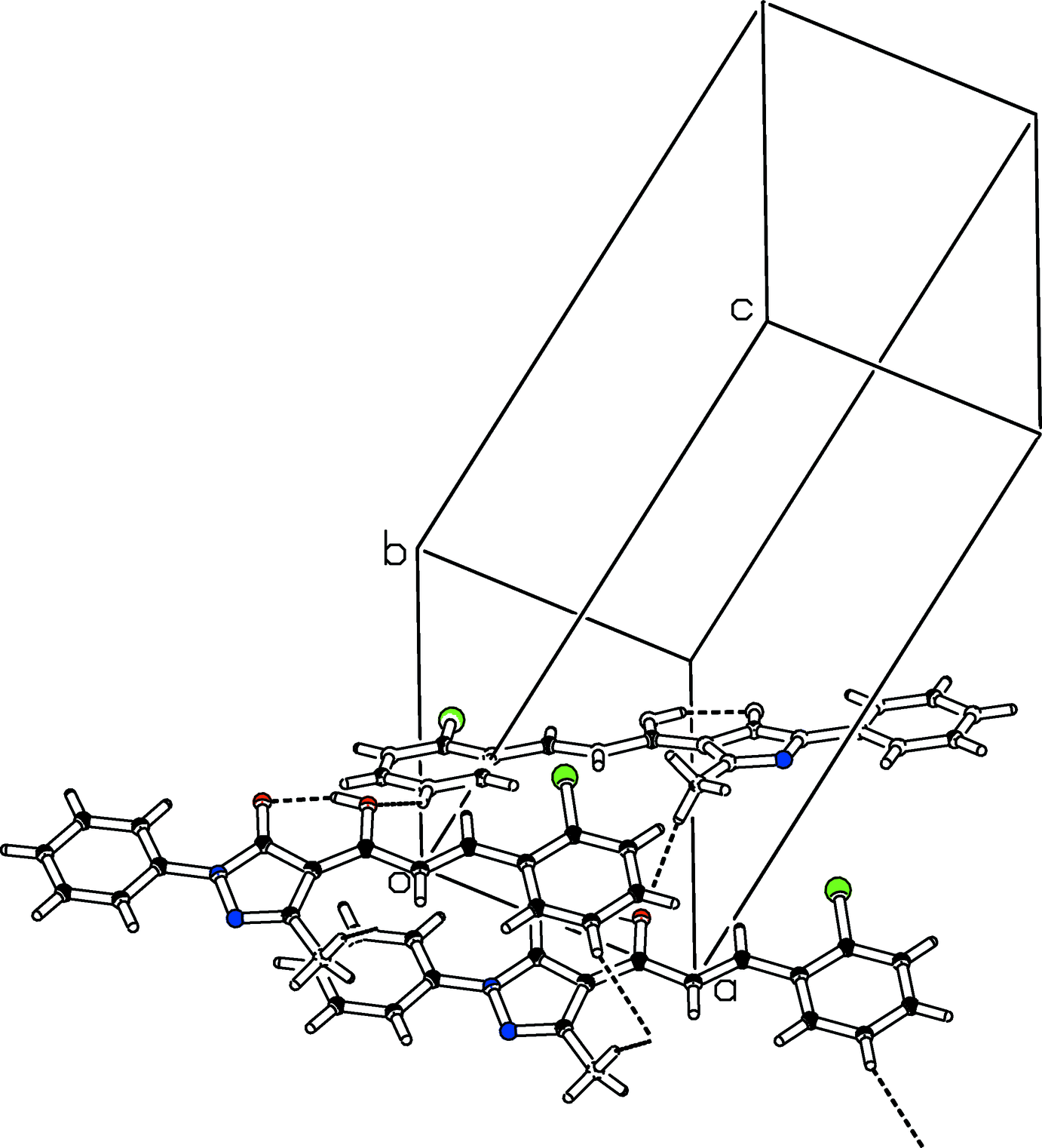

Supplement: Supplementary file 5 [file e-71-0o407-fig2.tif]
